# Does Dopamine Depletion Trigger a Spreader Lexical-Semantic Activation in Parkinson's Disease? Evidence from a Study Based on Word Fluency Tasks

**DOI:** 10.1155/2017/2837685

**Published:** 2017-06-11

**Authors:** S. Zabberoni, G. A. Carlesimo, A. Peppe, C. Caltagirone, A. Costa

**Affiliations:** ^1^Department of Psychology, Niccolò Cusano University, Rome, Italy; ^2^IRCCS Fondazione Santa Lucia, Rome, Italy; ^3^Department of Systems Medicine, Tor Vergata University, Rome, Italy

## Abstract

It has been hypothesised that, in Parkinson's disease (PD), dopamine might modulate spreading activation of lexical-semantic representations. We aimed to investigate this hypothesis in individuals with PD without dementia by assessing word frequency and typicality in verbal fluency tasks. We predicted that the average values of both of these parameters would be lower in PD patients with respect to healthy controls (HC). We administered letter-cued and category-cued fluency tasks to early PD patients in two experimental conditions: the tasks were administered both after 12–18 hours of dopaminergic stimulation withdrawal (“OFF” condition) and after the first daily dose of dopaminergic therapy (“ON” condition). HC were also given the two tasks in two conditions with the same intersession delay as PD patients but without taking drugs. Results showed that in both OFF and ON treatment conditions PD patients did not differ from HC in word frequency or typicality. Moreover, in the PD group, no significant difference was found between the experimental conditions. Our results show that semantic spreading was not altered in the PD sample examined; this suggests that in early PD the functioning of the semantic system is relatively independent from the activity of dopamine brain networks.

## 1. Introduction

Parkinson's disease (PD) is frequently accompanied by cognitive deficits. These include dementia or mild cognitive impairment involving attention, executive functions, visual-spatial abilities, and episodic memory [1]. It has been reported that the functional and structural modifications that take place in the frontal-striatal and mesolimbic circuitries in PD are associated with these cognitive changes [2–4].

Increasing attention has been given to the functioning of the lexical-semantic system in PD. Some studies have documented reduced semantic priming in PD patients with respect to healthy controls; this suggests that these patients have delayed lexical/semantic activation [5, 6]. In this vein, there is evidence that PD patients' performance on priming tasks is affected significantly by dopamine withdrawal [7, 8]. In particular, in addition to confirming reduced priming in PD patients when they were taking levodopa relative to healthy controls, Angwin et al. [8] also showed the lack of any priming effect when PD patients were assessed in the OFF condition. In agreement with previous studies conducted in healthy subjects [9–11], these findings underline the neuromodulatory role of dopamine within the semantic network [12] and also suggest that the speed of activation in PD patients is related to the extent of dopamine depletion [13].

According to the spreading activation theory, in the lexical-semantic network, the activation of individual nodes spreads to neighbouring concepts according to a variety of connections and nodes features [14]. In particular, the strength of association between nodes within the network might be modulated by the frequency of words. Hence, the activation of low frequency words would require greater spreading than the activation of high frequency words because the latter would have more and stronger links with other words in the network [15]. For this reason, in a word fluency task (in which subjects are required to generate as many words as possible according to some phonological or semantic constraints), healthy subjects typically produce more frequent words first. In PD patients, dopaminergic depletion could lead to an alteration of the structure of the lexical-semantic system with a reduced activation threshold difference between high and low frequency words as a manifestation of a spreader, less strategic, and seemingly random activation of the lexical units [8]. Accordingly, in a word fluency task, PD patients might generate words with lower frequency of use than healthy subjects. This issue was directly investigated in two studies which, however, reported inconsistent results. Indeed, Foster et al. [16] found that, in a phonological word fluency task, PD patients who were on dopaminergic treatment generated words with a significantly lower frequency of use compared to healthy controls. Conversely, Herrera et al. [17] found no direct effect of the manipulation of dopamine therapy on word use frequency in that PD patients produced words of comparable frequency irrespective of whether or not they were taking dopaminergic medication.

The aim of the present study was to further investigate lexical/semantic spreading activation in PD patients without dementia and its relationship to dopamine treatment. For this purpose, in addition to frequency of use computed on words generated in a letter-cued fluency task, we also assessed the typicality of the words produced in a category-cued fluency task. Indeed, the use of word typicality is underpinned by the assumption that each semantic category contains some words that are more representative than others [18]. Similar to what we discussed for high use frequency words, which should have a lower threshold of activation than low use frequency words, in a category-cued fluency task, words representing highly typical exemplars of a certain category should have a greater probability of being recalled than words representing less typical exemplars [18, 19]. To the best of our knowledge, the typicality index has never been used to investigate lexical-semantic spreading in PD patients.

Here we directly investigated the effect of dopaminergic stimulation on lexical-semantic activation in PD by contrasting the frequency of use and the typicality of words generated in fluency tasks in a sample of PD patients after withdrawal from (OFF condition) and after taking (ON condition) dopaminergic medication. Consistent with the assumption that dopamine has a significant neuromodulatory role in the strategic search and generation of words and, conversely, that dopamine depletion results in spreader, less strategic activation of units in the lexical-semantic system [16], we predicted that in the OFF condition PD patients would generate words with reduced frequency of use during the letter-cued fluency task and words less typical in the category-cued fluency task. Taking dopaminergic medication (on condition) should result in normalisation or, at the very least, in a significant increase in the average values of use frequency and typicality of the generated words.

## 2. Materials and Methods

### 2.1. Subjects

Twenty PD patients and 18 healthy controls (HC) were enrolled in the study after they gave their written informed consent. All of the patients included in the study were consecutive outpatients who had been referred by their primary care physician to the Parkinson's disease ambulatory care facilities of the IRCCS Santa Lucia Foundation in Rome. The diagnosis of idiopathic PD was made by a neurologist according to the London Brain Bank criteria [21]. Exclusion criteria for PD patients included (i) disease duration ≥ 5 years, (ii) diagnosis of dementia based on clinical criteria [22] and confirmed by a Mini-Mental State Examination [23] score < 26, and (iii) presence of other neurological and/or psychiatric illnesses in the patient's clinical history.

The HC participants were volunteers recruited from the patients' relatives. Exclusion criteria for the HC group included (i) cognitive impairment based on the Mini-Mental State Examination score < 26, (ii) taking medication that affects the central nervous system, and (iii) neurological and/or psychiatric illnesses, traumatic head injury, or substance abuse in the subject's history.

All PD patients were taking daily doses of dopamine or a dopamine agonist; in particular, seven patients were taking only L-Dopa, six patients were being treated with pramipexole or ropinirole only, and the remaining seven patients were taking both L-Dopa and dopamine agonists (i.e., pramipexole or ropinirole). All the patients presented bilateral akinetic-rigid form of PD and they were good and stable therapy responders. The clinical and demographic characteristics of the two experimental groups are reported in Table 1. L-Dopa equivalent doses are also reported for the patients' group.

Based on their performance on the tests included in the neuropsychological screening battery [24], 15 PD patients had only executive deficits, three patients had executive and episodic memory disorders, and the two remaining patients had visual-constructive apraxia.

### 2.2. Experimental Procedure

#### 2.2.1. Tasks

PD patients and HC were given letter-cued (phonemic fluency) and category-cued (semantic fluency) tasks. The experimental procedures were administered by an expert neuropsychologist.

In the* letter-cued word fluency task*, the subject has to generate as many words as possible that begin with a specified letter in three different trials, each lasting 60 seconds. Two versions of the task were created. In one version, the letters to be used to generate words were “A,” “F,” and “S.” In the other version, the letters to be used were “C,” “E,” and “L.”

Word use frequency was computed for each generated word according to normative values in the COLFIS corpus of Italian words [25].

In the* category-cued word fluency task*, the subject has to say as many words as possible that belong to a specific taxonomic category in two different trials, each lasting 60 seconds. Also in this case, two versions of the task were created. In one version, the categories to be used in the two trials of the task were “Trees” and “Furniture” and in the second version “colours” and “animals.”

The typicality value was computed for each word according to the category norms corpus for the Italian language [18].

The administration order of the two tasks was phonemic fluency followed by semantic fluency. At the beginning of each task, a training trial was given to be sure the subjects understood the instructions. Participants were told not to use proper nouns, not to use the same word with a different ending (e.g.,* arancia*,* arancione*,* aranciata*), and not to conjugate verbs. In each trial, the number of legal words generated in 60 seconds was recorded. Accuracy in each task was the sum of the number of legal words generated in all trials.

In order to evaluate in more detail the pattern of words generated in the two fluency tasks (in particular, whether the participants in the two groups produced, as expected, more typical/frequent words first and less typical/frequent words later), in each subject, average word use frequency (for the letter-cued fluency task) and average typicality (for the category-cued fluency task) were computed separately for the first half and second half of the words produced in the different trials.

#### 2.2.2. Design

PD patients were submitted to the experimental tasks after they had taken a full dose of stable dopaminergic treatment for one month. They were assessed in two experimental conditions that were performed on different days, with an intersession interval of about one month. In the “OFF” condition PD subjects performed the experimental tasks in the morning after 12/18 hours of drug withdrawal [26]. In the “ON” condition they were examined 90–120 minutes after they had taken their first morning dose of levodopa and/or dopamine agonists. To determine the efficacy of the dopamine compounds in improving extrapyramidal symptoms, in both treatment conditions, PD patients were given the UPDRS-Part III [27].

The tests of the experimental battery were administered to PD patients in both OFF and ON therapy conditions. By contrast, HC were given the tasks in two different sessions, named “blue” and “green,” without any drug administration. The “blue” session was associated with the OFF condition and the “green” session with the ON condition. The order of the experimental conditions (OFF/blue versus ON/green) was counterbalanced across subjects.

#### 2.2.3. Statistical Analysis

Modification of the UPDRS in the PD group as a function of the treatment condition was analysed by means of a repeated measures ANOVA. The average number of words generated on the two fluency tasks was analysed by means of two-way mixed ANOVAs with Group (PD versus HCs) as between subjects variable and Treatment (ON versus OFF condition) as within subjects variable. Finally, data relative to use frequency and typicality of words generated in the letter and category word fluency tasks, respectively, were analysed by means of three-way ANOVAs with Group (PD versus HCs) as between subjects factor and Treatment (OFF/blue versus ON/green condition) and Half (first half versus second half of the generated words) as within subject factors.

## 3. Results

### 3.1. UPDRS

Confirming the beneficial effect of dopamine stimulation for extrapyramidal symptoms, the UPDRS scores of patients with PD decreased significantly (Table 1) passing from the OFF (M = 16.3; SD = 7.6) to the ON (M = 11.8; SD = 4.3) treatment condition (*F*(1,19) = 8.25; *p* = 0.01).

### 3.2. Letter-Cued Word Fluency Task

The average number of words generated in the phonological word fluency task by PD patients and HC (Table 2) did not differ and it was not influenced by PD patients assuming medication as demonstrated by nonsignificant main effects of Group (*F*(1,36) = 1.70; *p* = 0.20) and Treatment (*F*(1,36) = 0.25; *p* = 0.61) and the Group × Treatment interaction (*F*(1,36) = .48; *p* = 0.49).

**Figure 1:**
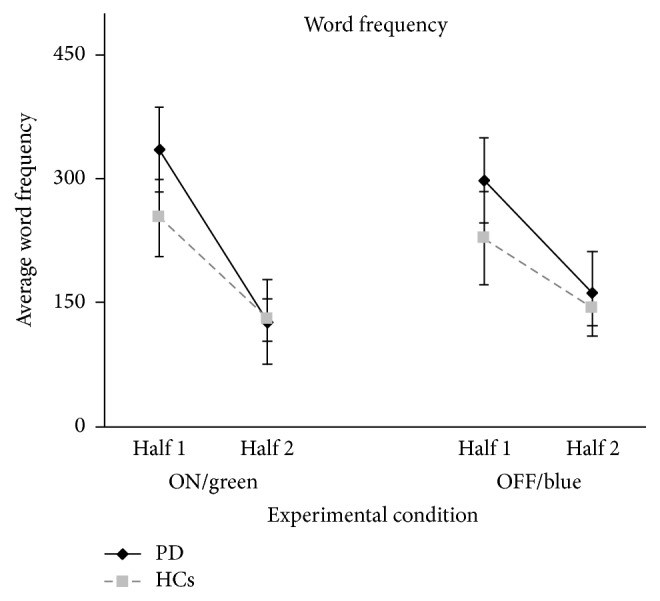
Average frequency of the words generated by PD and HC in both halves of trials of the phonemic fluency task, reported for both ON/green and OFF/blue experimental conditions.

The use frequency of words generated during the fluency task (Figure 1) also did not differ between groups and it was not influenced by dopamine stimulation. Indeed, only the main effect of Half was significant (*F*(1,36) = 8.83; *p* = 0.005), but the main effects of Group (*F*(1,36) = 0.69; *p* = 0.40) and Treatment (*F*(1,36) = 0.01; *p* = 0.93) as well as all the interactions (all *p* consistently >0.40) were not. In all subjects, word use frequency was higher for the words generated in the first half of the trial (M = 280.7; SD= 669.0) than for those generated in the second half (M = 139.9; SD = 184.1). Moreover, planned comparisons documented that PD patients generated words with comparable use frequency while taking dopaminergic medication (M = 230.97; SD = 467.19) and during treatment withdrawal (M = 229.6; SD = 739.7; *F* = .01; *p* = 0.97; Cohen's *d* = 0.002) and that a comparable decrease in use frequency passing from the first half to the second half of the trial was observed in words generated while patients were in the ON (M = 209.5; SD = 578.4) and the OFF (M = 136.8; SD = 255.0; *F* = .51; *p* = 0.48) treatment conditions (Figure 1).

### 3.3. Category-Cued Word Fluency Task

PD patients and HC did not differ either for the number of words generated in the two trials of the fluency task (*F*(1,36) = 0.18; *p* = 0.67). Furthermore, neither the Treatment factor (*F*(1,36) = 0.35; *p* = 0.56) nor the Group × Treatment interaction (*F*(1,36) = 0.35; *p* = 0.56) revealed significant effects, thus demonstrating that the average number of words generated by the PD patients was not affected when patients took L-Dopa medication (Table 1).

**Figure 2:**
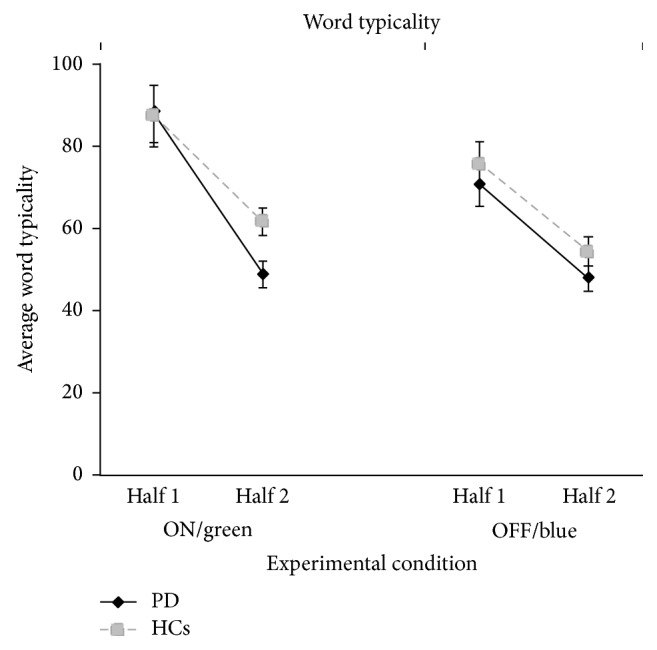
Average typicality of the words generated by PD and HC in both halves of trials of the semantic fluency task, reported for both ON/green and OFF/blue experimental conditions.

The average typicality of words also did not differ between groups and was not affected by the treatment condition (Figure 2). Indeed, also in this case, the Half main effect was significant (*F*(1.36) = 124.3; *p* < 0.001), whereas the Group (*F*(1.36) = 3.39; *p* = 0.07) and Treatment (*F*(1.36) = 3.20; *p* = 0.08) main factors and the second-order and third-order interactions were not (all *p* consistently >0.30). These data indicate that all subjects generated more typical words (within the semantic category) in the first (M = 80.7; SD = 52.5) than in the second (M = 53.3; SD = 30.0) half of the trials. Moreover, planned comparisons showed that the typicality of the words generated by PD patients when taking dopamine medication (M = 68.8; SD = 38.7) was not different from the typicality of words generated during medication withdrawal (M = 59.7; SD = 32.0; *F* = 1.64; *p* = 0.20; Cohen's *d* = 0.002) and that the average typicality of the generated word values decreased at the same rate passing from the first half to the second half in the ON (M = 39.3; SD = 57.2) and in the OFF (M = 22.9; SD = 47.9; *F* = .54; *p* = 0.46) treatment conditions.

## 4. Discussion

This study was aimed at investigating whether dopaminergic stimulation has a modulatory effect on the spreading activation of lexical-semantic representations in individuals with PD. In particular, we investigated whether reduced dopamine concentration results in increased spreading activation which could potentially influence strategic organization and retrieval of internal representations [16]. For this purpose, we administered a group of PD patients letter-cued and category-cued fluency tasks in two different pharmacological treatment conditions: (a) after a dopaminergic treatment wash-out period (“OFF” treatment condition) and (b) after they took their usual dopaminergic medication dose (“ON” treatment condition). We predicted that in the OFF condition PD patients, unlike HC, would show increased spreading activation documented by the generation of less frequent words in the letter-cued task and of less typical words in the category-cued task. Moreover, we predicted that taking dopamine medication (“ON” condition) would result in significantly less spreading of lexical-semantic activation, thus resulting in the generation of more frequent and more typical words.

Results did not confirm our predictions. Indeed, neither frequency nor typicality of the generated words differed between PD patients (in both OFF and ON treatment conditions) and HC. Moreover, no significant difference in these two parameters was found in the PD group in the two treatment conditions. To the extent that frequency of use and typicality of words generated in fluency tasks are behavioural indices of spreading activation within the lexical-semantic system [16], we can conclude that our PD sample did not present any significant alteration in this lexical-semantic system property and, therefore, that dopamine stimulation has no appreciable effect on the activation level of lexical-semantic representations.

Our findings are consistent with those of Herrera et al. [17]. These authors found no difference between PD patients and matched HC for frequency of use of words generated in a letter-cued fluency task and in two category-cued fluency tasks. The same study failed to reveal any effect of medication administration/withdrawal on the same indices in the PD group. However, these authors [17] found that, in an action-cued fluency task, PD patients in the OFF condition generated action words with greater use frequency than HC. Although the finding of an effect confined to the grammatical class of words is of interest in light of previous evidence of a special role of the frontal lobes in verb generation [28] and of a significant deficit of PD patients on verbs and action words [28], it is difficult to interpret. Indeed, in their PD sample, use frequency of words was not modulated by L-Dopa intake (i.e., there was no significant difference between PD patients in the ON and OFF treatment conditions). Moreover, the average frequency values in the group of PD patients and in HC could have been confounded by the different number of words generated (with higher values in the PD group possibly related to the lower number of words generated).

Therefore, taking together the above observations and the evidence that most PD participants in our study showed dysexecutive deficits, we argue that in the early stage of PD prefrontal lobe dysfunction does not affect processes involved in the maintenance of stable representations, such as those related to semantic knowledge. Coherently, the null effect of the therapy manipulation we found could be interpreted in the view that, in the early phases of PD, dopamine neurotransmission is mainly involved in the modulation of flexibility processes depending on the activity of the D2 dopamine receptors in the caudate nucleus [29–31] and does not affect the on-line processing of consolidated information.

However, our findings are at variance with those of Foster et al. [16]. These authors administered a letter-cued fluency task to groups of PD patients and matched controls and found that the frequency of use of words generated by PD patients was significantly lower than that generated by HC. One way of explaining these contrasting data is that the PD patients enrolled by Foster et al. [16] were in a more advanced stage of the disease compared to the PD patients who participated in our study (Foster et al.'s [16] study: mean disease duration = 6.8 years; mean UPDRS score = 32; our study: mean disease duration = 2.9 years; mean UPDRS score = 16.3). Therefore, we argue that the cortical regions responsible for the integrity of lexical-semantic processing are affected to a lesser extent in our PD sample than in the patients enrolled by Foster et al. [16]. Unfortunately, Foster et al. [16] did not manipulate dopamine treatment; thus we are unable to formulate any hypotheses about the role of dopamine stimulation on the effects they found.

Some limitations of the present study have to be discussed. First, likely because in the early stages of the disease, PD patients in the present study did not generate fewer words in the phonological and category-cued fluency tasks as compared to healthy controls, this could have reduced the possibility of finding significant effects of dopamine stimulation on the use frequency and/or typicality of produced words. Second, the PD patients were assessed while undergoing their usual dopamine therapy, which seemed to be quite heterogeneous as it includes levodopa and/or dopamine agonists. This could be another factor responsible of a lack of an effect of dopamine stimulation on spreading activation. Indeed, it is reported that the different molecules involved in dopaminergic compounds may have different effects on cognitive functions depending on their differential affinity with brain D_n_ receptors [32].

In conclusion, our results do not show a significant relationship between semantic spreading and dopamine stimulation in early-stage PD patients. However, also taking into account the above limitations, our findings might suggest the relative independence of the functioning of the semantic system and the activity of dopamine brain networks in the early stages of PD.

Finally, studies combining different paradigms (e.g., associative priming and verbal fluency) could be designed to further investigate the effect of dopamine treatment on lexical-semantic processing in PD.

## Figures and Tables

**Table 1:** Average (SD) of anagraphic data of experimental samples and clinical features of patient's group.

	PD (*n* = 20)	HC (*n* = 18)	*F* (df)	*p*
Age	66.7 (7.6)	67.9 (5.6)	0.3 (1,37)	0.57
Years of education	11.1 (4.2)	12.4 (3.4)	1.0 (1,37)	0.31
MMSE (raw score)	26.5 (0.45)	29.4 (0.76)	5.6 (1,37)	0.23
H&Y (range) [20]	2.5–3	—	—	—
Disease duration	2.9 (1.9)	—	—	—
UPDRS “ON”	11.8 (4.3)	—	8.2 (1,19)	0.01
UPDRS “OFF”	16.3 (7.6)	—		
L-Dopa equivalents	352.1 (138.5)	—	—	—
Therapy duration (years)	1.7 (0.6)			

**Table 2:** Average (SD) number of words generated by PD patients and HC in the letter-cued and category-cued fluency tasks. Note that PD patients performed the tasks in two distinct pharmacological conditions (ON versus OFF L-Dopa treatment), whereas HC took no drugs prior to either task session.

	Letter-cued		Category-cued	
	PD (*n* = 20)	HC (*n* = 18)	PD (*n* = 20)	HC (*n* = 18)
ON L-Dopa/green	26.7 (10.2)	31.2 (7.2)	21.9 (5.6)	21.6 (5.8)
OFF L-Dopa/blue	28.5 (10.7)	30.9 (8.6)	20.1 (8.3)	21.6 (4.5)
